# Breaking the “idling dilemma” of donated medical equipment: a TDABC-based analysis of health policy failure and a pathway to reform

**DOI:** 10.3389/fpubh.2026.1816383

**Published:** 2026-06-02

**Authors:** Heming Zheng, Yunmei Fang, Wenfu Pan

**Affiliations:** 1 School of Accounting, Guizhou University of Finance and Economics, Guiyang, Guizhou, China; 2 School of Medicine, Yangpu Hospital, Tongji University, Shanghai, China

**Keywords:** health inequity, health resource waste, medical equipment allocation policy, policy evaluation, sustainability, time-driven activity-based costing

## Abstract

**Objective:**

This study addresses the widespread idling and premature scrapping of donated medical equipment in grassroots and remote areas, revealing a key policy flaw: the current system incentivizes “equipment quantity and value” while systematically ignoring the essential “operation and maintenance (O&M) service ecosystem” needed for long-term usability.

**Methods:**

Using a Time-Driven Activity-Based Costing (TDABC) model, we analyzed a representative case—donating portable ultrasound devices to a high-altitude county hospital with a 5-year bundled service—to precisely quantify hidden long-term O&M costs for the first time.

**Results:**

The total present value (TPV) of 5-year O&M costs amounts to CNY 324,397.89, accounting for 30.19% of the overall total investment of CNY 1,074,397.89. Sensitivity analysis indicates that this proportion fluctuates between −4.15 and +27.84%. Travel and logistics alone exceed 70% of O&M costs, underscoring how geographic barriers erode policy effectiveness through high support expenses.

**Conclusion:**

Long-term O&M is a critical yet overlooked constraint; its underestimation is a critical factor likely associated with a “donate-and-abandon” model, potentially leading to resource wastage resources. We advocate shifting health policy from “equipment delivery” to “sustainable service capacity building,” proposing a life-cycle cost–based framework to institutionalize O&M costs and improve equity and efficiency in health resource allocation.

## 1 Introduction

In the context of addressing urban–rural health disparities and advancing universal health coverage, improving access to essential medical equipment in primary care and remote settings has become a critical health policy priority worldwide (1, 2). Governments, enterprises, and various sectors of society have actively responded through extensive donations, but this well-intentioned initiative has often fallen into the “high-input, low-impact” implementation dilemma: a large number of devices are quickly left idle after delivery, turning into “silent assets” due to a lack of maintenance, training, and consumables (3, 4). This not only results in a severe waste of public resources but also substantially undermines the capacity of primary healthcare services, thereby exacerbating health inequalities in the recipient regions.

This contradiction highlights the systemic bias in the design and evaluation of current medical equipment resource allocation policies. The existing policy framework and incentive mechanisms excessively emphasize visible outcomes on the “input side”—such as the quantity of donations, asset value, and coverage—forming an orientation that prioritizes “hardware delivery over capacity building” (5, 6). In practice, this value orientation manifests as institutional barriers at three levels:

At the policy formulation level, evaluation indicators overemphasize quantitative dimensions such as “coverage rates,” while neglecting quality metrics like equipment utilization and service accessibility (7); At the resource allocation level, fiscal funds primarily support equipment procurement, with a lack of institutional safeguards for sustained investments in subsequent maintenance, training, and other ongoing needs; At the implementation and oversight level, there is an absence of tracking and evaluation mechanisms for the effectiveness of equipment management throughout its entire lifecycle (8, 9). The policy consequence is the simplification of healthcare resource allocation into a one-time equipment transfer, while severely neglecting the operational ecosystem necessary to sustainably transform equipment into healthcare service capacity. This ecosystem includes multiple interconnected components such as technical support networks, talent development systems, consumable supply chains, and regional collaboration platforms (10). From the perspective of the entire lifecycle of health technology management, policy focus is concentrated on the “procurement” phase, with a lack of institutional support and financial guarantees for critical subsequent stages such as “usage, maintenance, and renewal.” This indicates a sharp decline in policy effectiveness once equipment delivery is completed.

The underlying issue lies in the widespread lack of life-cycle cost awareness in policy formulation. Especially in geographically remote and environmentally challenging areas, the long-term maintenance of equipment involves exorbitant and often unpredictable costs for technical support, personnel travel, and logistics (11, 12). Take high-altitude regions, for instance. Equipment maintenance not only involves routine servicing and repairs but must also account for the special impact of high-altitude environments on equipment performance, the cost of acclimatization for technical personnel, and the risks of logistical delays due to extreme weather, among other complex factors. These hidden costs constitute a significant portion of the equipment's original value, yet they are seldom factored into budget planning or policy evaluations. In the absence of precise cost measurement tools and institutional constraints, this hidden long-term expenditure is systematically underestimated by all stakeholders, eventually turning into an unassumed “sunk cost,” directly leading to equipment shutdown. This bias in cost perception exists not only among donors but is also prevalent among recipient institutions and local management bodies, creating a “collective blind spot” among all parties involved (8).

In this context, there is a need for analytical approaches that improve the transparency of hidden costs and incorporate empirically informed cost parameters. This study innovatively introduces TDABC. By developing a detailed analytical model for a “5-year bundled service for ultrasound equipment donated to high-altitude remote county hospitals,” it overcomes the limitations of traditional cost accounting methods. The model breaks down complex operation and maintenance activities into measurable activity units, enabling precise tracking and attribution of various resource consumptions. This approach effectively identifies cost drivers, distinguishes between fixed and variable costs, and provides granular evidence to support policy optimization (13, 14).

To ensure empirical rigor, the parameters for this TDABC model—including time equations, capacity cost rates and other related parameters—are derived from semi-structured interviews with domain experts. While this study focuses on a specific case of portable ultrasounds in high-altitude regions, it is framed as an exercise in analytical generalization rather than empirical generalization. The objective is not to provide a statistically representative cost average for all medical devices, but to utilize this “revelatory case” to expose the underlying mechanisms of health policy failure. By applying TDABC with empirical parameters to an extreme geographical context, we provide a theoretical and methodological framework that can be generalized to evaluate the fiscal sustainability of medical aid in other resource-constrained settings globally.

Based on this model, this study aims to achieve three core policy analysis objectives:

(1) To accurately measure the underestimated economic scale of long-term operation and maintenance services; (2) To analyze the cost structure and reveal the mechanisms through which policy factors such as geographical accessibility and equipment reliability impact sustainability; (3) To construct an evidence-based policy evaluation framework, promoting the transformation of health resource allocation from a “device delivery-oriented” approach to a “service capacity building-oriented” approach, thereby providing actionable reform pathways for systematically addressing idle equipment issues and enhancing policy effectiveness.

## 2 Methods

### 2.1 Study design

This study adopts a qualitative data-informed approach. Data derived from semi-structured interviews with domain experts are used to inform a TDABC modeling framework. The aim is to systematically examine the full lifecycle costs and their TPV required to ensure the sustained use of donated medical equipment. To achieve this, the TDABC model translates the objective of “sustained equipment use” into a structured resource consumption framework, enabling the identification and quantification of key cost components. This approach helps to make visible the gap between the full lifecycle costs of equipment ownership and the costs currently recognized in policy and budgeting practices. All key model parameters—including time, cost and other related parameters—are based on field data collected through interviews, with median values used for parameter setting.

### 2.2 Data collection

To improve the empirical grounding of the model, primary data were collected through semi-structured interviews. A total of 12 domain experts participated in the study, including maintenance engineers, physicians from the case hospital, and distributors. All participants were involved in equipment deployment, maintenance, or use. They participated in interviews to provide supporting data for model development and parameter specification. Due to confidentiality considerations, the identities of institutions are not disclosed. A sample of the interview guide has been provided in the supplementary materials.

### 2.3 Case basis and construction logic

This study examines a government-led medical equipment donation scenario in a resource-constrained setting. In the context of China's DRG/DIP payment reform, the case involves the deployment of three portable ultrasound devices (total fair value: 750,000 CNY) to a county hospital in Tibet, China, accompanied by a 5-year bundled service arrangement provided by the manufacturer. The analytical focus is placed on the lifecycle service costs and their TPV required to sustain equipment functionality over this period.

The selection of portable ultrasound deployment in high-altitude regions as the primary case follows the logic of a “revelatory and extreme case”. While the absolute cost figures are context-specific, this case reflects the “last-mile” challenges in health resource allocation. The extreme geographical barriers (altitude >4,000 m) and logistical bottlenecks serve as a “stress test” that magnifies the systemic flaws of current procurement-heavy policies—flaws that are often obscured in more accessible urban settings. Thus, the case serves as a platform for analytical generalization, where the identified mechanisms of cost formation can be projected onto other resource-constrained healthcare environments.

Within this context, the analysis focuses on the distinction between upfront capital expenditure and ongoing operational and maintenance requirements. While procurement costs are typically visible in budgeting and reporting processes, lifecycle service costs are often embedded in contractual arrangements and may not be fully incorporated into long-term financial planning. The model therefore aims to provide a structured representation of these cost components, rather than to evaluate policy performance directly.

To operationalize this analysis, the model follows a “resources → activities → cost objects” logic, identifying the resources and activities required to ensure sustained equipment operation over a 5-year period. A hybrid cost allocation strategy is adopted. For labor-intensive activities, the TDABC framework is applied through time equations. For non-time-driven expenditures, such as single trip cost, independently values (e.g., 6,500 CNY per trip) are incorporated to reflect contractual realities. The model includes not only explicitly defined service activities (e.g., installation, inspections), but also prerequisite (e.g., logistics) and contingent processes (e.g., return-to-factory repairs), ensuring a comprehensive representation of lifecycle service requirements.

### 2.4 Resource group identification and capacity cost ratio calculation

To establish a systematic costing framework, the model first identifies the primary resource groups involved in the service commitment: field service engineers and remote support agents. The Capacity Cost Rate (CCR)—representing the cost per minute of usable capacity—is the fundamental driver of the model. As shown in Equation 1, the CCR is calculated by dividing the total annual cost (including salaries, benefits, and high-altitude stipends for field engineers) by the annual effective working minutes:


$$\begin{array}{l} CCR_{k}=\frac{Total\;Annual\;Cost_{k}}{Annual\;Effective\;Working\;Minutes_{k}} \end{array}$$ (1)

Where *k* represents the resource group (field service engineers or remote support agents). *TotalAnnual cost* represents cost of field service engineer or remote support agents involved in high-altitude operations (including salary, high-altitude allowances, and training expenses). Remote technical support costs do not include high-altitude allowances.

### 2.5 Activity definition and cost allocation

**Figure 1:**
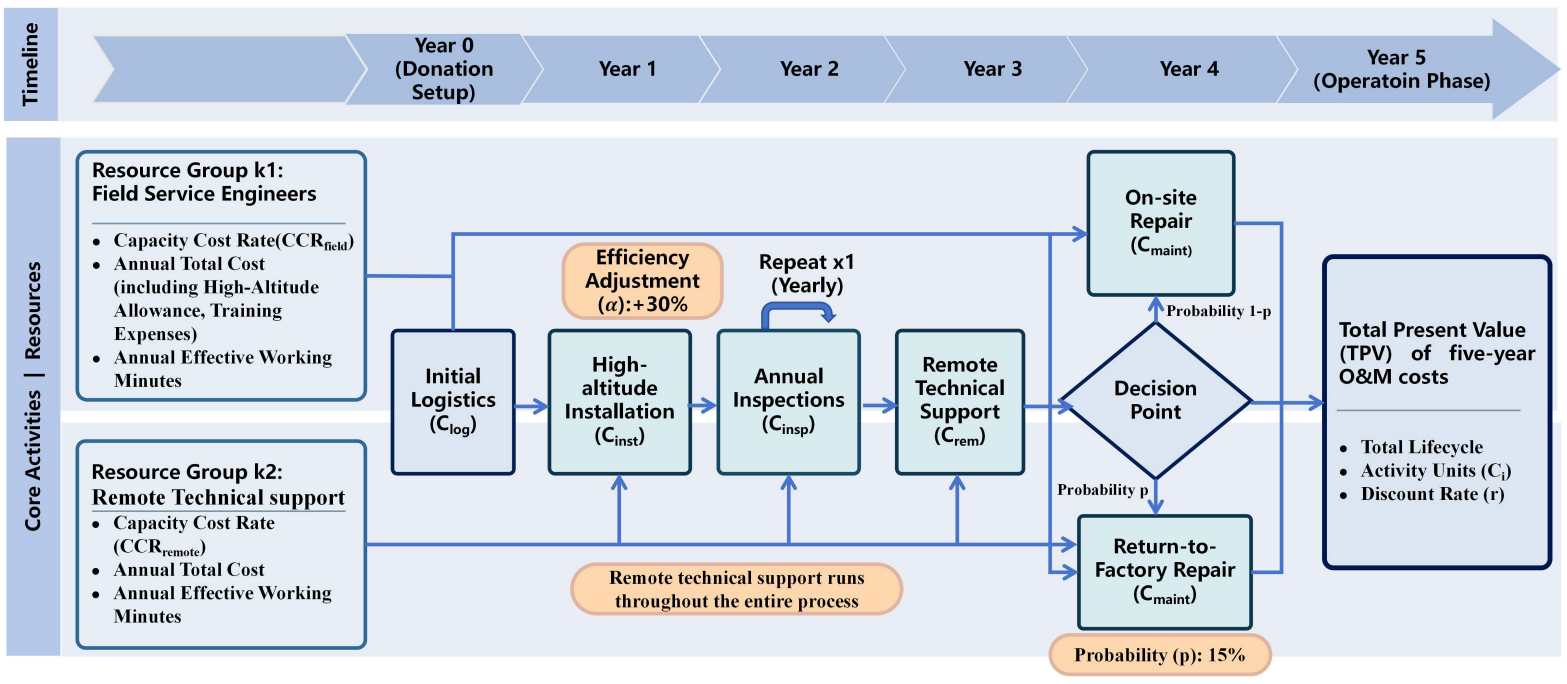
Analytical framework and process map of the TDABC model across the 5-year service lifecycle.

The TDABC model defines six core activities that span the 5-year service lifecycle. To ensure transparency and structural rigor, the cost (*C*) for each activity is formalized through specific equations, (see Figure 1 for the logic flow):

(1) Initial logistics (*C*_log_): this activity represents the fundamental logistical baseline. It is a one-time fixed cost incurred at Year 0 (CNY 15000), specifically covering the non-labor expenses required for the secure transportation of medical equipment from facility to high-altitude recipient sites.
(2) High-altitude installation (*C*_*inst*_): the installation phase accounts for both physiological and environmental constraints. To quantify this, an efficiency adjustment factor is applied to the standard labor duration (Equation 2):

$$\begin{array}{l} C_{inst}=T_{inst}\times \left(1+\alpha \right)\times CCR_{field}+C_{fixed\text{\_}trav} \end{array}$$ (2)

Where **T**_**inst**_ represents the benchmark time for equipment setup, while the coefficient **α** (set at 30%) serves as the efficiency adjustment factor to account for productivity loss due to physiological limitations in extreme high-altitude environments. The variable **CCR**_**field**_ denotes the capacity cost rate of field engineers. **C**_**fixed**_**trav**_ represents a fixed budgetary allocation for single trip cost.
(3) Annual inspections (*C*_*insp*_): these represent recurring preventive maintenance tasks (Once a year). The lifecycle cost for this activity is aggregated over the service horizon as shown in Equation 3:

$$\begin{array}{l} C_{insp}=\overset{5}{\underset{t=1}{\sum }}\left(T_{insp}\times CCR_{field}+C_{fixed\text{\_}trav}\right)\; \end{array}$$ (3)

Where **T**_**insp**_ is the cumulative annual time consumed per inspection task (cumulative time including **α**).
(4) Remote technical support (*C*_*rem*_): this captures the labor cost of technical consultation provided via telecommunication networks (Equation 4):

$$C_{rem}=\;\left(T_{rem}\times CCR_{remote}\right)\times N_{1}$$ (4)

Where *N*_1_ represents the total number of remote support instances over the 5-year service period, including routine remote assistance, *T*_*rem*_ signifies the time dedicated to remote technical support per session, and *CCR*_*remote*_ the capacity cost rate of remote technical support.
(5) Integrated maintenance (*C*_*maint*_): this comprehensive formula integrates the costs of both on-site repairs and return-to-factory repairs (Equation 5), dynamically weighted by the return-to-factory rate (*p*):

$$\begin{array}{l} C_{maint}=N_{2}\;\times \;[\left(1-p\right)\times \left(T_{repair}\times CCR_{field}+C_{fixed\_trav}\right)\\ \;+p\times (0.08\times V_{fair}+L_{round}+C_{fixed\_trav}+T_{repair}\\ \;\times CCR_{field})] \end{array}$$ (5)

In this comprehensive equation, *N*_2_ denotes the estimated total number of maintenance instances over the 5-year service period, and *p* represents the return-to-factory rate (15%). For on-site repairs, *T*_*repair*_ denotes the standard technical labor time (including **α**). For factory returns, the model incorporates a repair charge set at 8% of the equipment's fair value 0.08 × *V*_*fair*_ to account for component replacement, alongside *L*_*round*_for round-trip logistics.
(6) In the final stage of the structured TDABC model, the costs from all five defined activities are aggregated to determine the Lifecycle Total Cost (*TC*_*lifecycle*_). This metric represents the total nominal economic commitment required to sustain the equipment's functionality over the 5-year service horizon. The accumulation is formalized as shown in Equation 6:

$$\begin{array}{l} TC_{lifecycle}=\left(C_{\log}+C_{inst}\right)+\left(C_{insp}+C_{rem}+C_{maint}\right) \end{array}$$ (6)

This equation synthesizes the one-time initial setup expenditures at Year 0 (*C*_log_+*C*_*inst*_) with the cumulative operational outflows over the subsequent 5 years, providing a foundational baseline for further economic valuation. The benchmark values of all key parameters introduced in the above equations are summarized in Table 1 to ensure transparency and reproducibility.

**Table 1:** Key parameters of the TDABC model.

Category	Parameter	Symbol	Benchmark value	Description rationale
Financial	Discount rate	*r*	8%	Semi-structured interview
	Equipment fair value (3 devices)	*V*_*fair*_	750,000 CNY	Semi-structured interview
Capacity cost	CCR (field engineer)	*CCR*_*field*_	4.44 CNY/min	396,000 CNY/89,280 min
	CCR (remote support)	*CCR*_*remote*_	1.34 CNY/min	120,000 CNY/89,280 min
Time & efficiency	Installation time	*T*_*inst*_	312 min	1.3 × 240 min
	High-altitude efficiency reduction	α	30%	Semi-structured interview
	Annual inspection time	*T*_*insp*_	234 min	60 min × 3 devices × 1.3
	Unit repair time (On-site)	*T*_*repair*_	234 min	180 min × 1.3
	Unit remote technical support time	*T*_*rem*_	15 min	Semi-structured interview
Operational	Initial logistics cost	*C*_log_	15,000 CNY	Semi-structured interview
	Total number of remote support instances	*N*_1_	75 time	(1.5 × 3 devices × 5 years) × 2 + 2 × 5 years × 3 devices
	Total number of maintenance instances	*N*_2_	22.5 failure	1.5 × 3 devices × 5 years
	Return-to-factory rate	*p*	15%	Semi-structured interview
	Round-trip factory logistics	*L*_*round*_	30,000 CNY	*C*_log_× 2
	Single trip cost	*C*_*fixed*_*trav*_	6,500 CNY	Semi-structured interview

All parameters are informed by semi-structured interviews with domain experts.

CNY, Chinese yuan.

### 2.6 Calculation of cost present value

To accurately assess the economic value of the service commitment assumed at the donation date, this study applies the discounted cash flow method. All future activity costs are discounted back to the donation date (Year 0) using a benchmark discount rate of 8%, which corresponds to the median value derived from our semi-structured interviews with domain experts. In practice, costs incurred at the service initiation phase—specifically for initial logistics and high-altitude installation and commissioning—occur in Year 0 and are included at their nominal value. Recurring or probabilistic expenditures, such as annual preventive inspections, remote technical support, and the estimated costs for on-site and return-to-factory repairs derived from probability models, are discounted to their present value as annuities based on their scheduled timing. The total present value of the service commitment liability is calculated as the sum of the present values of all individual activity costs. This aggregate present value comprehensively represents, from a financial standpoint, the binding future economic obligation undertaken now of donation. As shown in Equation 7:


$$\begin{array}{l} TPV=\left(C_{\log}+C_{inst}\right)+\overset{5}{\underset{t=1}{\sum }}\frac{\frac{C_{insp}+C_{rem}+C_{maint}}{5}}{{\left(1+r\right)}^{t}}\; \end{array}$$ (7)

The *TPV* is the total present value of 5-year O&M costs to quantify the comprehensive economic liability assumed by the donor at the time of equipment deployment, of which *C*_log_ and *C*_*inst*_ represent the non-discounted initial investments at Year 0, specifically referring to the one-time cross-regional logistics expenses and the high-altitude installation costs.

### 2.7 Sensitivity analysis design

To further validate the robustness of the valuation results and pinpoint critical risk drivers, this study establishes a systematic five-dimensional sensitivity analysis framework to measure the effect of variations in key input parameters across plausible ranges (15, 16): (1) Single Trip Cost variation (5,000–8,000 CNY), capturing the influence of geographic and operational-environment shifts on on-site service expenses (2) Discount Rate range (6%−10%), testing the sensitivity of present-value outcomes to changes in cost-of-capital and risk-premium assumptions; (3) Annual Equipment Failure Rate range (1–2 failures per unit per year), evaluating how uncertainty in device reliability propagates into maintenance-activity costs; (4) Return-to-factory Rate range (10%−20%), gauging the effect of maintenance tactics and fault complexity on the incidence of high-cost factory returns; (5) Maintenance Cost range (15,000–25,000 CNY), accounting for variability owing to part replacement and intricate working conditions. By conducting multi-parameter and cross-scenario analysis, the study not only quantifies the overall potential variation in the service-liability present value but also specifically tracks changes in a pivotal ratio: the present value of total service costs relative to the fair value of the donated equipment (750,000 CNY). This methodology aims to elucidate the relative influence of distinct risk factors on the liability scale, thereby furnishing a robust quantitative basis for prudent accounting estimates, management decision-making, and ESG-oriented risk disclosure.

## 3 Results

### 3.1 Capacity cost rate

The calculated capacity cost rates are 4.44 CNY/min for field engineers (annual cost 396,000 CNY range:276,000–516,000 CNY) and 1.34 CNY/min for remote support personnel (annual cost 120,000 CNY range:96,000–144,000 CNY), based on an annual effective working time of 89,280 min (248 working days × 8 h/day × 75% efficiency ratio × 60 min). These figures reveal that the core cost of sustaining equipment availability in high-altitude regions lies not in the devices themselves, but in continuous human and technical support. Yet, the current health resource allocation system, which evaluates performance primarily based on “asset delivery volume,” systematically excludes this critical cost component—which ultimately implies service success or failure—from both budgeting and performance assessment.

### 3.2 Total service cost and total present value of the program

Based on the TDABC framework applied in this analysis, the calculation incorporates the following key operational and cost parameters (Table 1): a fixed single trip cost of 6,500 CNY, based on empirically informed parameters derived from field data annual failure rate of 1.5 incidents per device, an initial logistics cost of 15,000 CNY per dispatch, an installation duration of 4 h, an average remote support interaction of 15 min, a preventive maintenance session of 60 min, an on-site repair window of 3 h, a 30% productivity adjustment for high-altitude working conditions, a 15% likelihood of returning equipment to the factory for repair, and a factory repair charge set at 8% of the equipment's fair value.

The total cost of the 5-year all-inclusive service commitment is 400,464.18 CNY, with a present value of 324,397.89 CNY (discount rate 8%). Combined with the equipment's fair value of 750,000 CNY, the total economic value reaches 1,074,397.89 CNY, of which the service present value accounts for 30.19%. Quantitative evidence suggests that ensuring the continuous usability of a donated device over 5 years requires additional operation and maintenance resources equivalent to 31.9%. This 30.19% “hidden service liability” represents a critical accounting dimension entirely absent from current charitable donation accounting standards and public procurement policies, perpetuating the widely accepted but misleading notion that “donating equipment equates to enhancing capacity.”

### 3.3 Cost composition per service

**Figure 2:**
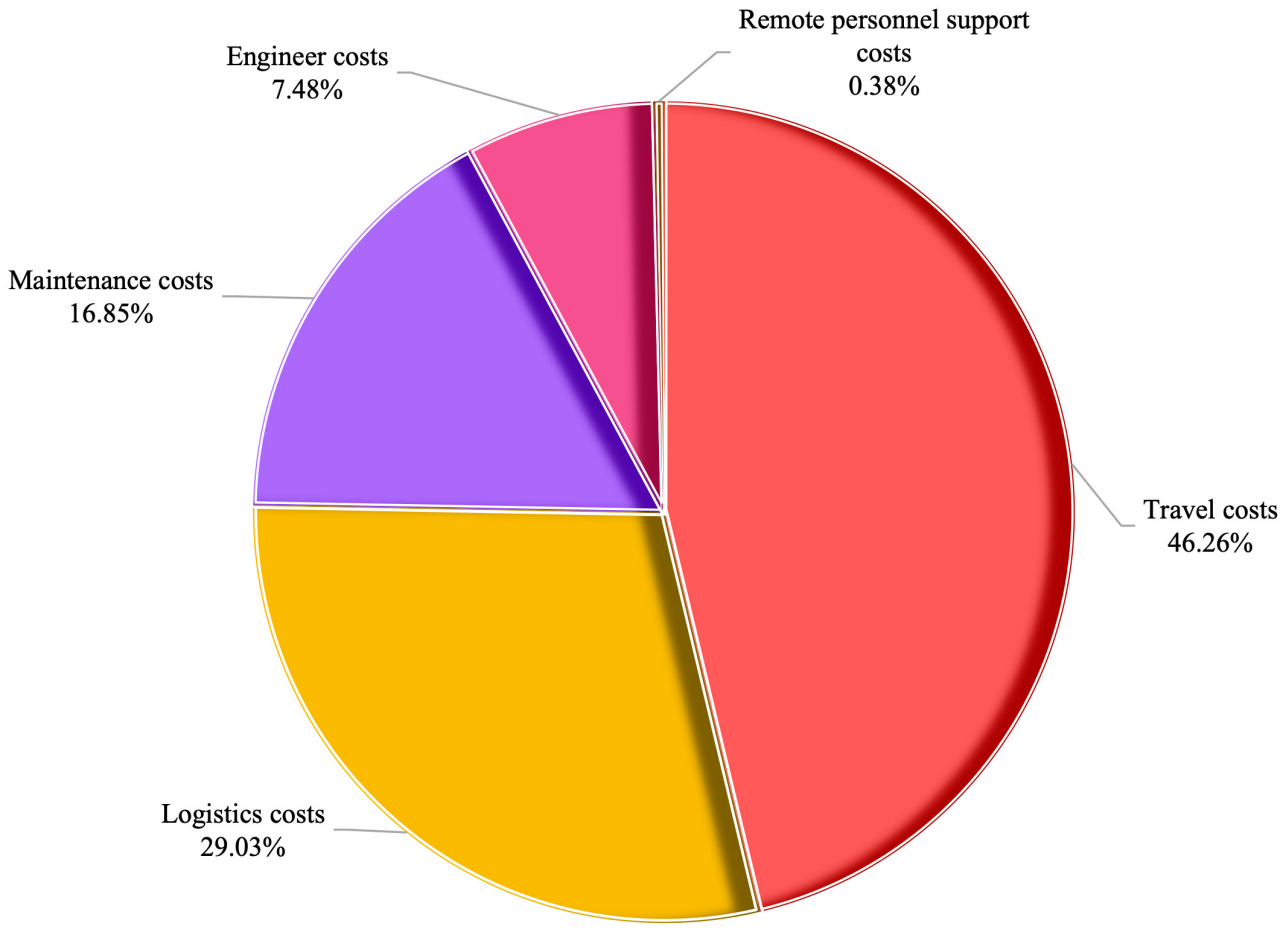
Cost structure of lifecycle service for donated medical equipment.

The cost structure (Figure 2) reveals that logistics costs amount to 116,250 CNY (29.03%) and travel costs total 185,250 CNY (46.26%), together accounting for 75.29% of the total operational costs. In contrast, maintenance costs account for 16.85% (67,500 CNY), while engineer costs (7.48%) and remote support costs (0.38%) represent only a marginal fraction of the budget. Initial analysis of this composition correlates geographical distance with significant economic barriers to service accessibility. Consequently, equipment donation policies intended to promote health equity may have their intended effects offset—or even reversed—by the inherent geographical disadvantages of recipient regions. This suggests that unless a “high-altitude geographical cost factor” is institutionalized within fiscal compensation mechanisms, such policies risk exacerbating hidden disparities in regional service capacity. However, interpreting these logistical expenses solely through the lens of geographical determinism provides an incomplete picture. To deepen the interpretative framework, these cost structures should be understood as symptoms of broader socio-technical and contextual vulnerabilities: (1) Human Capital Deficit: The heavy reliance on travel costs (46.26%) serves as a proxy for local technical absence. The inability to train or retain resident biomedical engineers in grassroots hospitals necessitates a total dependency on external dispatch, converting what could be routine internal maintenance into expensive inter-provincial logistics. (2) Market Monopoly and Vendor Lock-in: In remote contexts, the absence of a competitive third-party repair market leaves hospitals with minimal bargaining power. Consequently, they are locked into Original Equipment Manufacturer (OEM) service networks, where travel premiums are dictated by suppliers rather than negotiated through market competition. (3) Infrastructural Vulnerability: The frequency of logistics-heavy interventions is further exacerbated by hostile environmental contexts. Remote high-altitude regions often suffer from unstable power grids and lack climate-controlled storage, which may accelerate equipment degradation and trigger expensive factory returns more frequently than in urban centers.”

### 3.4 Sensitivity analysis results

The sensitivity analysis (Table 2) indicates that the TPV is most sensitive to variations in the annual equipment failure rate, with changes reaching approximately ±27.84%. This suggests that equipment reliability is a key determinant of uncertainty in long-term operation and maintenance (O&M) costs. From a policy perspective, this highlights the importance of strengthening quality assurance mechanisms at the pre-deployment stage. Insufficient consideration of environmental adaptability and durability may be associated with increased downstream maintenance burdens, suggesting the potential value of establishing formal high-altitude applicability and reliability assessment standards. The return-to-factory rate (±13.85%) also shows a notable impact on *TPV*. This indicates that the design of maintenance and repair pathways—specifically the balance between on-site servicing and centralized repair—may have important cost implications. From a policy perspective, this suggests the need to optimize maintenance system design, for example by improving local service capacity or refining repair allocation mechanisms. Single Trip Cost (±10.62%) further reflect the influence of geographical constraints on service delivery. This highlights that spatial accessibility and distance-related factors may systematically affect cost structures. It suggests that uniform funding and allocation mechanisms may not fully capture such spatial heterogeneity, and that more context-sensitive approaches—such as regionalized service networks or differentiated subsidy schemes—could be considered. By contrast, financial parameters, including the discount rate (−4.70 to +5.11%) and maintenance cost, show relatively limited impact. This indicates that the overall cost pattern is relatively robust to financing assumptions. From a policy perspective, this suggests that adjustments to financing conditions alone may have a comparatively smaller influence on long-term cost variability. Overall, these findings suggest that operational factors—particularly equipment reliability and service delivery design—may play a more prominent role in shaping lifecycle cost outcomes than financial parameters, with each key parameter broadly corresponding to distinct policy-relevant dimensions, including quality assurance, maintenance system design, service network configuration, and financing conditions.

**Table 2:** Parameters for one-way sensitivity analysis.

Variable parameters	Parameter benchmark value	Reasonable test range	Total present value fluctuation range (benchmark: 324,397.89 CNY)	Variation range
Annual equipment failure rate	1.5 failures/unit·year	1.0−2.0 failures/unit·year	234,087.82–414,707.96 CNY	−27.84% to +27.84%
Return-to-factory rate	15%	10%−20%	279,479.9–369,315.88 CNY	13.85% to +13.85%
Single trip cost	6,500 CNY	5,000–8, 000 CNY	289,958.03–358,837.75 CNY	10.62 to +10.62%
Discount rate	8%	6%−10%	309,149.5–340,985.22 CNY	−4.70% to +5.11%
Maintenance cost	20,000	15,000–25,000 CNY	310,922.5–337,873.29 CNY	−4.15% to +4.15%

Under multifactor scenarios, the present value of service liability fluctuates only within a range of −12.15 to +13.01%. This result indicates that as long as core operational parameters (such as failure rate and on-site repair rate) are effectively controlled, long-term service costs can be accurately predicted and incorporated into medium-term fiscal planning. This provides empirical support for reforming the current “one-time funding, no follow-up responsibility” donation model—policy should shift from “equipment delivery and acceptance” to “5-year availability commitments,” making service costs explicit, contractual, and budgeted.

It should be emphasized that the foregoing all results are derived from a TDABC-based framework parameterized through expert-informed data; consequently, these findings should be interpreted as strategic indications of cost structures rather than direct empirical observations of retrospective accounting.

## 4 Discussion

This study examines the 5-year lifecycle service costs and their TPV associated with the deployment of portable ultrasound devices in high-altitude remote areas. The findings suggest that current medical equipment donation frameworks may place greater emphasis on hardware provision than on long-term operation and maintenance requirements. This pattern is consistent with prior studies highlighting the limited sustainability of technology deployment in resource-constrained settings (17, 18).

A key insight from the analysis is that operational expenditures—particularly those related to logistics and travel—constitute a substantial share of TPV. This cost structure suggests that sustaining the continuous operation of advanced equipment may pose challenges for grassroots healthcare institutions, even when equipment is physically available. In this context, the findings resonate with existing literature describing the phenomenon of underutilized or non-functional donated technologies in low-resource environments (19, 20). Such patterns may be interpreted as reflecting a form of “symbolic donation,” where the provision of equipment does not necessarily translate into effective service capacity.

Importantly, these patterns do not appear to be attributable solely to geographical isolation. While physical distance is associated with higher logistical costs, the results indicate that such pressures may be reinforced by broader contextual constraints, including limited local technical capacity and the structure of service provision systems. This suggests that cost burdens emerge from the interaction between geographical conditions and institutional arrangements, rather than from distance alone. In this sense, approaches that focus primarily on hardware provision may not fully account for the absence of local technical ecosystems or the limited accessibility of repair services in underserved regions.

Building on these observations, the study further relates to a set of structural tensions in current policy frameworks—namely, an emphasis on quantity over quality in goal-setting, procurement over operation and maintenance in resource allocation, and delivery over sustainability in accountability mechanisms. These patterns correspond to what may be described as a “triple policy gap,” which appears to be more pronounced in geographically constrained regions, where logistical barriers amplify the consequences of limited service capacity.

These observations suggest that improving the long-term effectiveness of donation programs may require greater integration of operational and maintenance considerations into policy design. This may involve aligning procurement decisions, service arrangements, and evaluation mechanisms with the full lifecycle requirements of medical equipment (21, 22).

### 4.1 Analytical generalization and contextual applicability

The evidence presented in this study offers significant analytical generalization for global health policy. Although the data are derived from high-altitude regions in China, the underlying mechanism—whereby “hidden” logistics and labor costs eclipse the initial equipment donation value—is applicable to other resource-constrained or geographically isolated settings, such as archipelagic nations in Southeast Asia, rural Sub-Saharan Africa, or Guizhou Province in China. Furthermore, the TDABC framework applied here can be extended beyond ultrasound equipment to other complex medical technologies (e.g., X-ray or ventilators) that require specialized maintenance, providing a universal tool for assessing the fiscal sustainability of medical aid projects.

### 4.2 Policy recommendations

The findings of this study point to potential imbalances in current donation frameworks, where a predominant focus on equipment delivery may not fully account for long-term operational sustainability. Based on the model results, several policy recommendations are outlined to improve the alignment between resource allocation and service capacity.

The sensitivity of *TPV* to equipment failure rates reflects the relevance of pre-deployment screening conditions in shaping downstream maintenance requirements. In this context, establishing a life cycle cost-based evaluation and certification system—requiring donation projects to include operation and maintenance cost estimations and funding guarantee plans over a defined service period—may provide a more structured basis for project approval (23, 24). At the same time, the structural and recurrent nature of operation and maintenance costs is associated with ongoing financial demands that may not be fully addressed under existing funding arrangements. Dedicated fiscal mechanisms, such as earmarked transfer payments or maintenance funds, may offer more stable support for equipment use in primary healthcare settings.

In addition, the observed relationship between equipment characteristics and long-term costs highlights the role of reliability, durability, and maintainability in shaping cost structures. Incorporating these factors into procurement and approval standards, alongside technical specifications, may improve alignment with local service conditions, particularly in relation to modular design and service accessibility (25).

From a service delivery perspective, the pattern of maintenance costs reflects the importance of local technical capacity. Where such capacity is limited, reliance on distant repair channels may become more pronounced. The development of regional technical service platforms, such as shared maintenance centers or integrated service systems, may help support more balanced service provision.

Finally, the comparison between cost structures and evaluation practices indicates that current frameworks may place greater emphasis on equipment availability than on functional performance. Incorporating outcome-oriented indicators, such as utilization rates, mean time to repair, and user satisfaction, may contribute to a more comprehensive assessment of equipment effectiveness (26, 27).

### 4.3 Limitations and future research

Of course, this study also has certain limitations. Although the model parameters are informed by primary data collected through semi-structured interviews, they remain subject to uncertainty due to the limited sample size and the context-specific nature of the case (28); this may affect the precision of the cost estimates, and the reported values should therefore be interpreted as indicative rather than exact representations of real-world costs.

The study's focus on a single technology (portable ultrasound) within a specific geographical context may limit the empirical generalizability of the exact cost ratios (e.g., 30.19%). However, following the principles of analytical generalization, this study aims to provide a structured process-mapping approach and to identify underlying cost-driver mechanisms, rather than to establish universally applicable numerical results. Future research could apply this TDABC-based evaluation framework to diverse medical modalities and varied socio-economic contexts to further examine the consistency of these patterns.

While the TDABC model is parameterized using empirical data from field interviews, the projected 5-year outcomes represent model-based economic inferences rather than longitudinally observed empirical causalities. As such, the findings should be interpreted as indicative of potential cost structures under specified assumptions.

Finally, this study primarily focuses on economic costs and does not quantify broader social and health consequences, such as clinical misjudgments, patient attrition, or erosion of trust in the system resulting from equipment unavailability. Incorporating these dimensions in future research may provide a more comprehensive understanding of the implications of equipment donation programs.

Future research should deepen efforts in two key areas: First, from a policy perspective, exploring reasonable cost-sharing mechanisms for operation and maintenance funds within multi-level governance structures, as well as sustainable operational models for regional technical service platforms. Second, from an academic perspective, conducting full-cycle cost comparison studies across regions and multiple device types to identify the operational and maintenance sensitivities of different technologies in varying environments. Third, integrating implementation science frameworks to investigate which organizational mechanisms (such as regional technical support centers or integrated clinical-engineering teams) can effectively reduce operational costs and enhance equipment utilization. The potential of digital health tools (such as remote quality control and AI-assisted fault diagnosis) in bridging geographical gaps also merits in-depth research.

## 5 Conclusion

This study utilizes a TDABC-based framework parameterized through semi-structured interviews with domain experts to examine lifecycle cost structures associated with medical equipment donation. The analysis provides quantitative insights into a potential gap in current health policy frameworks, particularly regarding the limited incorporation of long-term operation and maintenance (O&M) costs. The objective of these findings is to illustrate that the chronic “idling dilemma” observed in grassroots health systems may be closely associated with a disconnect between hardware delivery and service sustainability.

Our findings icate that these expert-informed estimations, which place the O&M burden at approximately 30.19% of the total lifecycle value, may reflect a structural financial gap that is not always explicitly accounted for in existing allocation mechanisms. It should be noted that these results, while informed by empirical expert inputs, represent indicative cost structures rather than direct retrospective accounting data.

Within this analytical context, the results suggest that a shift in policy orientation—from an equipment-delivery focus toward a more service-capacity-oriented approach—may warrant consideration. Incorporating lifecycle costing into policy design, including funding arrangements and evaluation frameworks, may contribute to improving the sustainability of medical equipment deployment. Such an approach may help enhance the likelihood that donated technologies in remote regions are effectively utilized and maintained over time.

## Data Availability

The data analyzed in this study is subject to the following licenses/restrictions: the data are not publicly available due to privacy. Requests to access these datasets should be directed to jzhwz325@163.com.
