# Supplementary material for: Breaking the “idling dilemma” of donated medical equipment: a TDABC-based analysis of health policy failure and a pathway to reform
Source: Front Public Health. 2026 Jun 2;14:1816383. doi: 10.3389/fpubh.2026.1816383 (PMC13269226; doi:10.3389/fpubh.2026.1816383)
Supplement: Supplementary file 1 [file Data_Sheet_1.pdf]

Semi-structured Interview Guide

I. Interview Objective

To examine the full lifecycle operation and maintenance (O&M) costs of donated medical equipment in high-altitude remote regions. To enhance the empirical grounding and real-world relevance of the model inputs, expert interviews were conducted with the following objectives:

1. To collect practitioner-informed inputs for key TDABC model parameters, including human resource costs, equipment reliability indicators, maintenance activity characteristics, logistics-related costs, and other relevant parameters.

2. Cost Driver Identification: To develop an in-depth understanding of how high-altitude geographical conditions influence medical equipment O&M costs, and to clarify the structure and composition of different cost components.

3. Policy Insight Exploration: To gather practitioner perspectives on the limitations of current medical equipment donation policies and potential areas for improvement.

2. Interview Method

Item	Description
Interview Type	Semi-structured interview.
Interview Format	In-person or video conference; approximately 45–60 minutes per session.
Recording Method	Written notes taken by the interviewer after obtaining verbal informed consent from the respondent; no audio recording.
Ethical Handling	All interview records are anonymized. Identifiable information such as names and institutional affiliations is removed; only institutional type and professional background are retained for data quality documentation purposes.
Data Usage	Used solely for parameter elicitation for the TDABC model in this study; no other uses.

3. Interview Respondents

3.1 Expert Selection Criteria

Criterion	Requirement
Years of experience	At least 5 years of relevant professional experience.

Operational experience	Direct frontline experience in the deployment, installation, or maintenance of medical equipment in high-altitude regions (above 4000 meters) of Western China.
Participant background	Maintenance engineers, Physicians from the hospital, and Distributors.
Position level	Mid- to senior-level professionals with relevant experience in medical equipment operation, maintenance, or management.

3.2 Respondent Information (Anonymized)

Item	Content
Expert Code	XXXXX
Participant Background	□Engineer□ Physician (hospital) □Distributors Other: ____
Position/Role	________________
Years of Experience	________________
Primary Service Region	□Tibet Plateau □ Yunnan-Guizhou Plateau □ Other high-altitude/remote area: ____
Number of high-altitude projects involved	□ 1–5 □ 6–15 □ 15 or more

4. Interview Questions

Part 1: Background Perception and Qualitative Judgment of Cost Drivers (Approx. 10 minutes)

Purpose: To establish the respondent's overall perceptual framework regarding the unique characteristics of high-altitude O&M work, providing contextual grounding for subsequent parameter elicitation.

Question 1
Based on your experience, what are the main challenges in operating and maintaining medical equipment in high-altitude or remote regions?.

Question 2
Among these challenges, which single factor do you believe contributes most significantly to driving up total O&M costs? Please briefly explain why.

Part 2: Elicitation of Core TDABC Model Parameters (Approx. 30 minutes)

Purpose: To obtain industry consensus values for key model parameters, item by item.

Final parameter values are based entirely on the respondent's independent professional judgment.

A. Human Resource Costs and Efficiency

Question 3
Based on your experience, what would be a typical range for the annual cost of a field service engineer involved in high-altitude operations (including salary, allowances, and related expenses)?

Question 4
Based on your experience, what would be a typical range for the annual cost of personnel providing remote technical support?

Question 5
In your experience, approximately how many hours per year are effectively available for direct service tasks, after accounting for travel, training, and administrative duties?

Question 6
How does working in high-altitude environments affect on-site work efficiency compared to lowland conditions? Please describe the typical difference based on your experience.

B. Equipment Reliability and Maintenance Activity Parameters

Question7
Based on your experience, for comparable projects involving the deployment of portable ultrasound devices in high-altitude remote settings (e.g., a three-unit bundled arrangement), what range would you consider representative of the overall fair value?

Question 8
Based on your experience, how frequently do portable ultrasound devices typically encounter failures when operating in high-altitude environments?

Question 9
For a typical on-site repair task, approximately how much time is usually required for diagnosis and repair after arriving on site?

Question 10
For routine preventive maintenance visits, approximately how much time is usually required for inspection and servicing (excluding travel time)?

Question 11
For remote technical support (e.g., phone or video guidance), approximately how much

time is typically required per session?

Question 12
Based on your experience, in what situations would equipment need to be returned to the factory for repair, and how common is this compared to on-site repairs?

Question 13
For return-to-factory repairs, how would you describe the typical cost level relative to the value of the equipment?

C. Logistics and Travel Cost Parameters

Question 14
Based on your experience, what are the main cost components involved in travel and logistics for a typical on-site service visit in high-altitude regions?

Question 15
In your view, how significant are travel and logistics costs compared to other components of total O&M costs?

D. Financial Parameter

Question 16
Based on your experience, what is the typical range of discount rates or expected returns used in long-term service or maintenance-related projects?

Part 3: Policy and Mechanism Exploration (Approx. 10 minutes)

Purpose: To obtain qualitative recommendations for policy reform from the perspective of frontline industry practitioners.

Question 17
In your view, what are the main reasons for the low utilization or idling of donated medical equipment in remote primary-care settings?

Question 18
What is your view on the feasibility of establishing regional shared O&M service centers or third-party maintenance platforms? What potential advantages or challenges do you see?

Question 19
Do you have any suggestions for improving the sustainability and effectiveness of medical equipment donation programs?

Part 4: Closing Confirmation (Approx. 5 minutes)

Question 20
Are there any additional factors that you consider important for understanding O&M costs in high-altitude regions that we have not discussed?

Question 21
Do you consent to the use of insights derived from this interview in an anonymized and aggregated form in this study?

5. Interviewer Record Sheet

Item	Content
Interview ID	202X-0XX
Interview Date	____ (Day) ____ (Month) __(Year)
Interview Duration	________ minutes
Interviewer Signature	________________
Post-Interview Supplementary Notes	
Data Entry Date	____ (Day) ____ (Month) __(Year)
